# Low grade appendiceal mucinous neoplasm mimicking an ovarian cyst: A case report

**DOI:** 10.1016/j.ijscr.2020.04.074

**Published:** 2020-05-07

**Authors:** Abdulaziz Omar Alghamdi, Mohammed Yousef Aldossary, Morshed Alsawidan, Shoukry AlBahar

**Affiliations:** a Department of General Surgery, Imam Abdulrahman Bin Faisal University, P.O. Box 1982, Al-Khobar, Dammam 31441, Saudi Arabia; b Department of General Surgery, King Fahad Specialist Hospital, Dammam, Saudi Arabia; c Department of General Surgery, Dammam Medical Complex, Saudi Arabia

**Keywords:** AM, appendiceal mucocele, MRI, magnetic resonance imaging, US, ultrasonography, AJCC, American Joint Committee on Cancer, CT, computed tomography, Appendix, Mucocele, Ovarian cyst

## Abstract

**Introduction:**

Appendiceal mucocele (AM) is a very rare neoplasm with an incidence of 0.2–0.3% of all appendectomy specimens. AM usually defined as an intraluminal dilation of the appendix in response to mucin accumulation. The preoperative diagnosis is difficult and can be misdiagnosed to any of the other differential diagnosis especially in female patients. If it is suspected, imaging modalities and colonoscopy can be helpful as a guide for the right surgical approach.

**Presentation of case:**

A 41-year-old lady who found to have an appendicular tumor discovered incidentally on magnetic resonance imaging (MRI) of the abdomen during a follow-up in the referral hospital for persistent right lower quadrant abdominal pain with enlarging right ovarian cyst for 2 years. MRI revealed a sizeable cyst measuring 7 × 4 × 3 cm in the right iliac fossa (RIF) region with high suspicion of an AM. The patient was managed with open right limited hemicolectomy. The patient was diagnosed as a well-differentiated low-grade AM neoplasm with stage 0 (pTis, pN0, M0) based on the 8th edition of the American Joint Committee on Cancer (AJCC) Staging System. The patient had an uneventful recovery and advised for regular follow-up of at least 5 years.

**Discussion:**

AM is a very rare neoplasm. The imaging modalities that can be diagnostic methods are the abdominal ultrasonography (US), transvaginal US, abdominal computed tomography (CT) and abdominal MRI. Fine needle aspiration must be avoided as the risk of perforation is high that will lead to dissemination of the mucinous material causing a serious complication called pseudomyxoma peritonei. Laparotomy is superior on laparoscopy as the risk of perforation is lower in laparotomy.

**Conclusion:**

The presentation of AM is usually not specific, because of its anatomic position. Physicians should consider it in the differential diagnosis of persistent enlarging ovarian cyst or adnexal mass.

## Highlights

• Appendiceal mucocele defines as an intraluminal dilation of the appendix in response to mucin accumulation.
• This condition overall is very rare with a 0.2–0.3% incidence of all appendectomy specimens.
• It's more common in the females with a 4 to 1 ratio.
• Appropriate diagnosis and management of appendiceal mucocele prevent complications such as pseudomyxoma peritonei.

## 1 Introduction

AM defines as an intraluminal dilation of the appendix in response to mucin accumulation. This condition overall is very rare with a 0.2–0.3% incidence of all appendectomy specimens [[1], [2], [3]]. It’s more common in females with a 4 to 1 ratio and more prevalent in individuals above 50 years old [4,5]. Patients with AM usually are asymptomatic and they get diagnosed incidentally through radiological intervention or intra-operatively for any other co-existing condition. But some patients experience symptoms like abdominal pain in the RIF region, RIF palpable mass, gastrointestinal bleeding, weight loss, nausea, and vomiting [4,5]. The only definitive and curative treatment of AM is surgical resection. The management of AM is a comprehensive process starting from suspicion of AM, proper workup, right diagnosis, choosing the proper treatment then follow-up. This case report is in line with the SCARE criteria [6].

## 2 Presentation of case

**Fig. 1:**
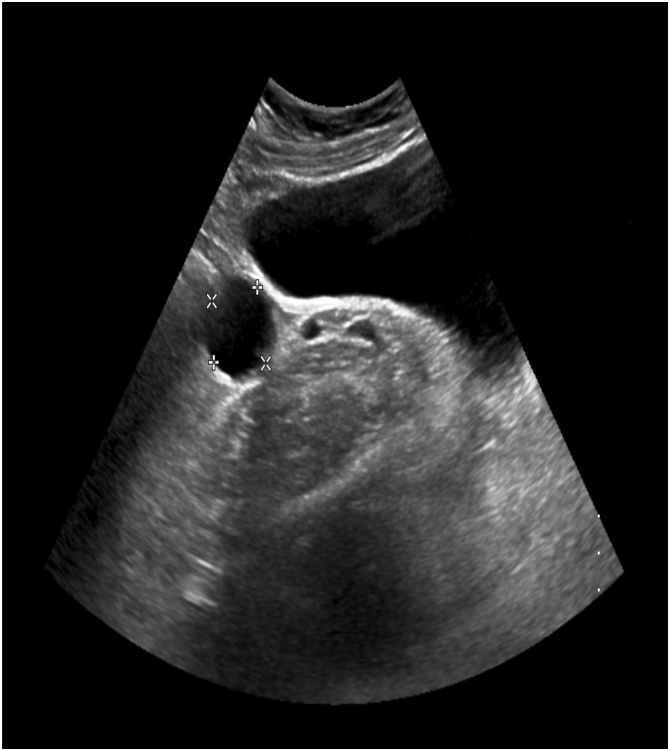
US abdomen showed both ovaries are normal in volume, and echogenicity bilateral multiple follicles are seen. The right ovary shows cyst measuring about 3 × 2.87 cm.

**Fig. 2:**
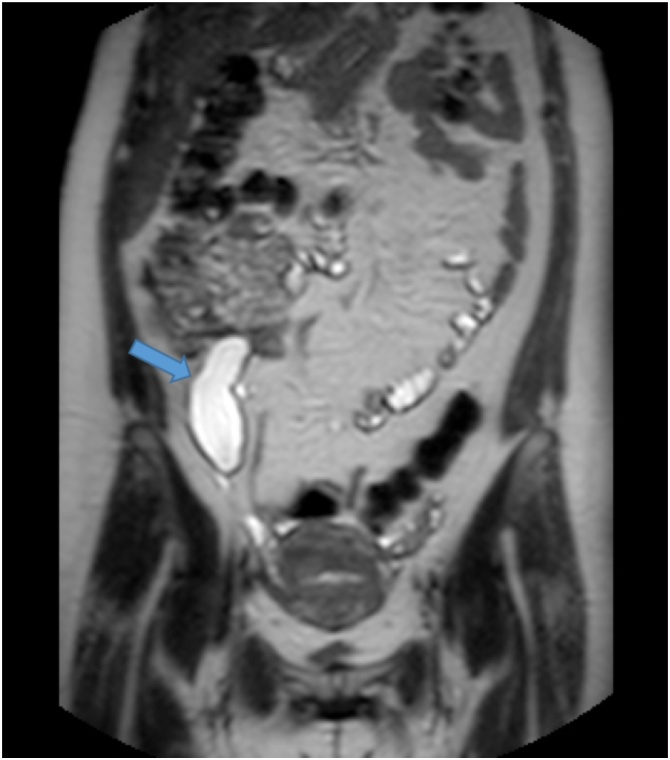
MRI of the abdomen showed: Sizable cyst-like measuring 7 × 4 × 3 cm in the right iliac fossa region, very close to the cecum and away from the right ovary and adnexa.

**Fig. 3:**
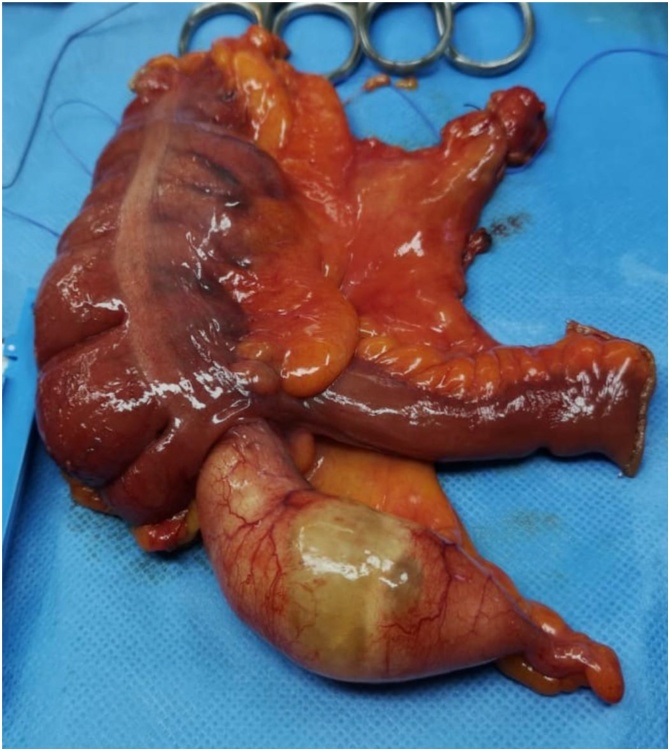
Mucinous neoplasm growing in the appendix (intraoperatively).

A 41-year-old married female, known to have a hiatal hernia with gastroesophageal reflux disease. The patient found to have an appendicular tumor discovered incidentally on MRI during a follow-up in the referral hospital for persistent right lower quadrant abdominal pain with enlarging right ovarian cyst for 2 years. The patient had no history of irregular bowel movements or abnormal changes in the stool. The patient denied any history of anorexia, weight loss, and jaundice. Her menstrual periods were regular. Past medical and surgical histories including family history for malignancy were unremarkable. The patient underwent serial US in the referral hospital which revealed a cystic lesion in the right ovary measuring 3 × 2 cm increased up to 6 × 2.8 cm (Fig. 1). Due to her persistent pain and increase in the size of the ovarian cyst in the US, the patient underwent MRI which revealed a sizable cyst measuring 7 × 4 × 3 cm in the RIF region, away from the right ovary and adnexa with no fat stranding, or mesenteric lymph nodes enlargement (Fig. 2). The impression of an appendicular tumor was highly suspicious. Upon arrival at our hospital, the patient was well-nourished, in mild pain, and had no pallor or jaundice. Abdominal examination revealed mild tenderness with a vague fullness on deep palpation of the right iliac fossa. Laboratory examination demonstrated the following: haemoglobin: 10.9 g/dl, leucocyte count: 6.0 × 10^9^/L, haematocrit: 35.3%, and platelet count: 280 × 10^9^/L. The liver function test, renal function test, and the coagulation profile were all within normal ranges. Cancer antigen markers including carcinoembryonic antigen and carbohydrate antigen 19.9 were all within the normal range. Colonoscopy was done as a preoperative workup and was unremarkable. The patient underwent laparoscopic exploration which confirmed the presence of AM involving the base of the appendix, inseparable from the caecum. We did not find any synchronous neoplasms in the colon. The case was then converted to open exploration through a lower midline laparotomy incision which showed a cystic mass measuring 9 × 3 × 3 cm involving most of the appendix including the base inseparable from the caecum, sparing 2 cm tip with multiple palpable ileocecal mesenteric lymph nodes. The cystic lesion seems about to rupture at the center. A Limited right hemicolectomy with side to side ileocolic stapler anastomosis was performed. Gross examination revealed a dilated appendix with smooth shiny serosa and thin wall, and it is filled with thick mucus material (Fig. 3).

**Fig. 4:**
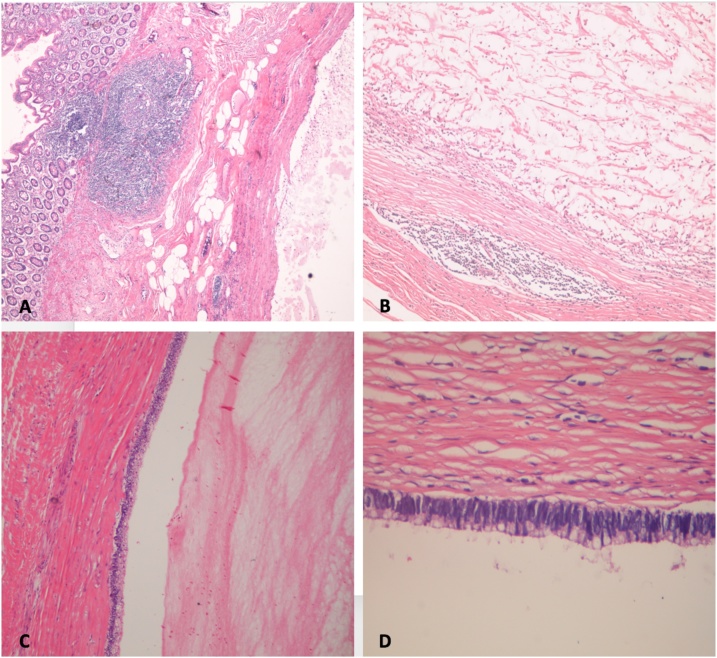
**A**: Hematoxylin and eosin staining (40×) revealed the lumen of appendix lined by unremarkable colonic mucosa with underlying lymphatic aggregates. There is a cystic dilatation space filled with acellular mucin. This cystic space starts replacing appendiceal mucosa epithelial lining, lamina propria, and settle in the muscularis propria without invasion. **B**: Haemotoxylin and eosin staining (100×) revealed no mucosa or lamina propria. There is epithelial denudation in the lack of acellular mucin. The neoplasm is rest on fibrotic stroma filled with lymphatic aggregates. **C**: Haemotoxylin and eosin staining (100×) revealed a lumen of mucocele contains acellular mucin and mucinous surface epithelium. **D:** Hematoxylin and eosin staining (400×) revealed an appendiceal mucosa is replaced by tall, columnar, non-ciliated cells, basal nuclei, and abundant intracellular mucin.

Histopathological examination revealed acellular mucin invading submucosa and muscularis propria, but not through the full thickness of the muscularis propria. All margins of resection were free from the tumor. Nine lymph nodes were examined and all were negative. No lymphovascular invasion. The patient was diagnosed as a well-differentiated low-grade AM neoplasm with stage 0 (pTis, pN0, M0) based on the 8th edition of the AJCC Staging System (Fig. 4A, B, C, and D). The diagnosis of a low grade appendiceal mucinous neoplasm was made. The postoperative recovery was uneventful, and there were no signs or symptoms of recurrence within the follow-up period of 1 year. The patient was advised to complete a follow-up period for at least 5 years.

## 3 Discussion

As AM is very rare, preoperative diagnosis is difficult and can be misdiagnosed to any of the other differential diagnoses especially in female patients. The diagnosis of AM is usually made in an acute setting (< 7.5%) and in a chronic setting (15–29%) [7]. If the AM is suspected, imaging modalities and colonoscopy can be helpful. The imaging modalities that can be diagnostic methods are the abdominal US, transvaginal ultrasound, abdominal CT, and abdominal MRI [7,8]. Usually, the US will show a cystic mass with variable echogenicity and sonographic layering within the cyst (called “onion skin sign”), and If the diameter of the appendix in the US is 15 mm or more, the sensitivity of 83% and specificity of 92% for the diagnosis of AM [8,9]. AM appears in CT as cystic, tubular or spherical, low attenuated structure [[7], [8], [9]]. Colonoscopy should be considered preoperatively if there is suspicion of AM. Usually, the colonoscopy will show a visible elevated area in the cecum with the orifice of the appendix in the center of it (volcano sign) and may reveal yellow discharge [8,9]. Fine needle aspiration must be avoided as the risk of perforation is high that will lead to dissemination of the mucinous material causing a serious complication called pseudomyxoma peritonei [9].

Previously, the AM was classified histologically into: simple mucocele, hyperplastic mucocele, mucinous cystadenoma and mucinous cystadenocarcinoma [7,9,10].

Nowadays, it is classified based on histopathological examination according to the degree of atypia into low-grade appendiceal mucinous neoplasms or high-grade appendiceal mucinous neoplasms [7,11].

The management of AM is a comprehensive process starting from suspicion of AM, proper workup, right diagnosis, choosing the proper treatment then follow up. The only definitive and curative treatment of AM is surgical resection. In the literature, there is no total agreement on the best surgical approach that can be done to treat the AM. Many circumstances and factors determine the proper surgical technique. The aim of surgery is to resect the mucocele properly and not to cause perforation and spillage of the content causing pseudomyxoma peritonei, a life-threatening complication with less than 45% of 10-years survival and less than 20% of 5-years survival [[10], [11], [12], [13]]. Laparotomy is superior on laparoscopy as the risk of perforation is lower in laparotomy. Appendectomy is enough in cases that in which appendicular base is not involved, the cecum is intact and the mucocele is not perforated. While right hemicolectomy is the recommended choice in case of involvement of the appendicular base, cecum, perforated mucocele, and positive lymph nodes [10,14,15]. There may be synchronous neoplasms with the AM and most frequently found in the colon [16]. However, it may be found in other locations such as breast, gallbladder, kidneys, thyroid, and ovaries. The recurrence rate of AM is approximately 5% and the relapse is more frequent between 12 and 24 months [16]. AM was found to be associated with adenocarcinoma of the colon in 19%–25% of the cases [17]. Based on that, all cases identified to have AM should be assessed for colon neoplasms. In our patient, we did not find any pathology during the colonoscopy performed pre-operatively. Also during the laparoscopic exploration, we didn't find any synchronous colon neoplasm. Postoperative, we advised the patient for regular follow-up of at least 5 years to detect any recurrence or relapse.

## 4 Conclusion

AM is a rare neoplasm in abdominal surgery. The abdominal US, abdominal CT, and abdominal MRI represent useful tools for diagnosis. However, the preoperative diagnosis is difficult and can be misdiagnosed. Appropriate diagnosis and management of AM prevent complications such as pseudomyxoma peritonei. All cases identified to have AM should be assessed for synchronous colonic neoplasms. Colonoscopic surveillance of patients for synchronous colonic neoplasms is warranted.

## Declaration of Competing Interest

The authors report no conflicts of interest.

## Sources of funding

This study did not receive any funding from governmental or private organizations.

## Ethical approval

This is a case report and it didn’t require ethical approval from ethics committee according to our institution.

## Consent

Written informed consent was obtained from the patient for publication of this case report and accompanying images. A copy of the written consent is available for review by the Editor-in-Chief of this journal on request.

## Author contribution

Study concept or design – AOS, MYD, SB.

Data collection – AOS, MYD, MS, SB.

Data interpretation – AOS, MYD, MS.

Literature review – AOS, MYD, MS, SB.

Drafting of the paper – AOS, MYD, MS.

Editing of the paper – AOS, MYD, SB.

## Registration of research studies

Not required.

## Guarantor

Mohammed Yousef Aldossary.

## Provenance and peer review

Not commissioned, externally peer-reviewed.
